# Serofast Syphilis Is Associated with Phospholipid-Dependent Coagulation Abnormalities and B-Cell Activation Following Treatment

**DOI:** 10.3390/ijms27114954

**Published:** 2026-05-29

**Authors:** Martyna Kiolbasa, Konrad Kaminiow, Damian Kadylak, Maciej Pastuszczak

**Affiliations:** 1 Clinical Department of Dermatology, Medical University of Silesia, 41-800 Zabrze, Poland; martynakiolbasa@gmail.com (M.K.); kaminiow.k@gmail.com (K.K.); 2 Department of Dermatology, Venereology and Allergology, Medical University of Gdansk, 80-214 Gdansk, Poland; damiankadylak@gmail.com

**Keywords:** syphilis, serofast, coagulation, autoimmunity, VDRL

## Abstract

A substantial proportion of patients treated for early syphilis fail to achieve the expected ≥4-fold decline in non-treponemal titers despite appropriate therapy. This serofast state remains a common clinical dilemma, and repeated antibiotic therapy is often ineffective. We hypothesized that persistent seroreactivity may reflect infection-associated immune dysregulation rather than ongoing infection. In this prospective study, 36 adults with early syphilis were treated with benzathine penicillin. At 6 months, 11 patients with inadequate serological response underwent cerebrospinal fluid evaluation; 3 with neurosyphilis were excluded. The remaining 33 patients were classified as serofast (n = 8) or serologically cured (n = 25). Eleven healthy individuals served as controls. Serofast patients demonstrated prolonged phospholipid-dependent coagulation assays compared with serologically cured individuals (all q_BH_ < 0.01; δ = 0.75–0.83). They also exhibited higher BAFF levels and B-cell counts at baseline and follow-up. Posttreatment VDRL titers strongly correlated with BAFF levels, B-cell counts, and coagulation parameters. After exclusion of neurosyphilis, persistent non-treponemal seroreactivity was associated with coordinated B-cell activation and phospholipid-dependent coagulation abnormalities, suggesting an infection-triggered immune phenotype rather than ongoing *Treponema pallidum* infection.

## 1. Introduction

The treatment of early syphilis relies on intramuscular benzathine penicillin G, and to date, *Treponema pallidum* has shown no evidence of resistance to this therapy [1]. Despite its high efficacy, approximately 10–20% of patients fail to achieve an adequate serological response, defined as the absence of at least a fourfold decline in non-treponemal test titers within 6–12 months after treatment [2,3]. This condition, referred to as the serofast state, represents a common and clinically challenging scenario in the post-treatment management of syphilis.

The clinical significance of the serofast state remains uncertain. While it has traditionally raised concern for persistent infection or treatment failure, several observations challenge this interpretation. A proportion of serological non-responders are subsequently diagnosed with neurosyphilis, indicating ongoing infection requiring intensified therapy [4]. However, in the majority of cases, neurosyphilis is excluded, and repeated antibiotic treatment does not improve serological outcomes [5,6]. These findings suggest that persistent non-treponemal reactivity may not necessarily reflect the presence of viable spirochetes but could instead represent a host-driven immunological phenomenon.

Emerging evidence supports the role of immune dysregulation in the pathogenesis of the serofast state. In previous studies, we demonstrated that patients who remain serofast exhibit features consistent with altered immune regulation, including increased prevalence of antinuclear antibodies and signs of autoimmunity [7]. Similarly, other investigations have shown that serological outcomes in syphilis are influenced by host immune profiles, including impaired Th1 responses and enhanced immunoregulatory pathways [7,8]. Together, these findings suggest that the serofast state may reflect a dysregulated immune response rather than persistent infection.

Non-treponemal tests such as the Venereal Disease Research Laboratory (VDRL) and rapid plasma reagin (RPR) assays detect antibodies directed against cardiolipin-containing antigens, which are not specific to *T. pallidum*. These antibodies are well known to occur in autoimmune diseases, particularly systemic lupus erythematosus, where they are associated with antiphospholipid antibodies and lupus anticoagulant activity [9,10]. Importantly, in such conditions, non-treponemal test reactivity reflects autoantibody-mediated processes rather than infection.

Laboratory detection of lupus anticoagulant relies on phospholipid-dependent coagulation assays, including lupus anticoagulant–sensitive activated partial thromboplastin time (aPTT), dilute Russell viper venom test (LA1 and LA2), and mixing studies such as the PTT-LA mix [11,12]. These assays do not measure clotting factor deficiencies but instead identify functional interference caused by antiphospholipid antibodies, serving as indirect markers of autoimmune activity.

Interestingly, similar coagulation abnormalities have been reported in a range of infectious diseases. Transient lupus anticoagulant positivity and antiphospholipid antibodies have been described in COVID-19, HIV, and hepatitis C virus infection, often in association with systemic inflammation and immune activation [13,14,15,16]. These observations indicate that infections can trigger antiphospholipid antibody production and functional coagulation disturbances independent of classical autoimmune disease.

In parallel, increasing attention has been given to the role of B-cell–mediated immune activation in infection-associated autoimmunity. B-cell activating factor (BAFF), a key regulator of B-cell survival and differentiation, promotes the expansion of autoreactive B cells and the production of autoantibodies [17,18]. Elevated BAFF levels have been reported in both autoimmune diseases and chronic infections, suggesting that infection-driven BAFF signaling may contribute to dysregulated humoral immunity.

Given that non-treponemal tests primarily detect anticardiolipin reactivity and that phospholipid-dependent coagulation assays identify functional effects of antiphospholipid antibodies, we hypothesized that the serofast state may represent, at least in part, an infection-induced autoimmune phenomenon. Specifically, persistent non-treponemal seroreactivity could be driven by BAFF-mediated B-cell activation and the generation of antiphospholipid antibodies, leading to both sustained VDRL positivity and measurable disturbances in phospholipid-dependent coagulation assays.

## 2. Results

### 2.1. Patient Characteristics

A total of 36 consecutive patients with early syphilis were enrolled in the study. At baseline, blood samples were collected from all participants for serological, immunological, and coagulation analyses. All patients subsequently received standard therapy with a single intramuscular dose of benzathine penicillin G (2.4 million units).

At six months after treatment, 11 patients failed to achieve an adequate serological response, defined as the absence of at least a fourfold decline in VDRL titer compared with baseline. All of these patients underwent cerebrospinal fluid (CSF) examination to evaluate for neurosyphilis.

Neurosyphilis was diagnosed in 3 patients, who were excluded from further analysis. The remaining 8 patients, in whom central nervous system infection was excluded, were classified as the serofast group. The remaining 25 patients achieved an adequate serological response and were classified as the serologically cured group (non-serofast group).

Follow-up blood samples for immunological and coagulation analyses were obtained at six months from all patients included in the final analysis (n = 33). The two groups were comparable in terms of age, sex distribution, and disease stage, with similar proportions of secondary and early latent syphilis. No significant differences were observed in baseline hematological or inflammatory parameters between groups. Detailed demographic and laboratory characteristics are presented in Table 1.

### 2.2. Immune Parameters

**Figure 1:**
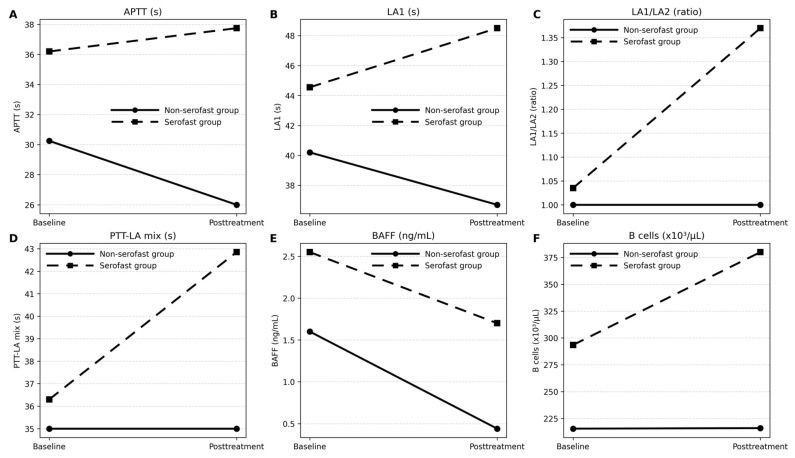
Longitudinal changes in phospholipid-dependent coagulation and immune parameters in serofast and non-serofast patients. Median values are shown at baseline (before treatment) and 6 months after therapy. Solid lines with circular markers represent the non-serofast group (n = 25), whereas dashed lines with square markers represent the serofast group (n = 8). (**A**) Activated partial thromboplastin time (aPTT-LA). (**B**) Dilute Russell’s viper venom time screening test (LA1). (**C**) LA1/LA2 ratio. (**D**) Phospholipid-dependent mixing test (PTT-LA mix). (**E**) Serum B-cell activating factor (BAFF) concentration (ng/mL). (**F**) Peripheral blood B-cell count (×10^3^/µL). Values are presented as medians. Baseline samples were obtained prior to antibiotic administration; posttreatment samples were collected 6 months after completion of therapy.

To explore potential immunological differences associated with the serofast phenotype, we compared serum BAFF levels and peripheral B-cell counts between groups. As summarized in Table 1 and illustrated in Figure 1E,F, patients in the serofast group exhibited consistently higher BAFF levels and B-cell counts than non-serofast individuals.

Median BAFF levels were significantly higher in the serofast group both at baseline and posttreatment (*p* = 0.03 and *p* < 0.001, respectively). Similarly, B-cell counts were elevated in serofast patients at baseline (*p* = 0.03) and remained higher after treatment (*p* = 0.001), indicating enhanced B-cell–related immune activity in individuals with persistent serological reactivity.

To evaluate whether treatment-associated changes differed between groups, a two-way model including group, time, and group × time interaction was applied. BAFF levels were significantly higher in serofast patients (*p* = 0.0017), and an overall decrease after treatment was observed (*p* < 0.001). However, the group × time interaction was not significant, indicating that the magnitude of BAFF change after treatment did not differ between groups.

A similar pattern was observed for B-cell counts, which were significantly higher in the serofast group (*p* = 0.018), whereas neither the overall time effect nor the group × time interaction reached statistical significance.

### 2.3. Phospholipid-Dependent Coagulation Profile

At six months post-treatment, patients in the serofast group demonstrated consistently prolonged phospholipid-dependent clotting times compared with those who achieved serological cure. As shown in Table 2 and Figure 1, median posttreatment values of aPTT, LA1, LA2, LA1/LA2 ratio, and PTT-LA mix were all higher in the serofast group.

Effect size analysis confirmed the strength and coherence of these findings across assays. All parameters showed large effect magnitudes (Cliff’s δ = 0.75–0.83), with longer clotting times in serofast patients, and all remained statistically significant after false discovery rate correction (q_BH_ < 0.01).

Although only two patients fulfilled formal ISTH criteria for lupus anticoagulant positivity, the overall prolongation of phospholipid-dependent clotting assays in the serofast group was consistent across all analytical methods (Table 2).

### 2.4. Associations Between Serological Activity, Immune Parameters, and Phospholipid-Dependent Coagulation Assays

To further explore potential links between immune activation and phospholipid-dependent coagulation abnormalities, correlation analyses were performed, including BAFF levels, B-cell counts, VDRL titers, and coagulation parameters measured at baseline and six months after treatment.

Significant associations were observed between immune activation markers and phospholipid-dependent coagulation assays (Supplementary Figure S2). In particular, posttreatment BAFF levels correlated with posttreatment aPTT, LA1/LA2 ratio, and PTT-LA mix, indicating that higher BAFF concentrations were associated with greater prolongation of phospholipid-dependent coagulation parameters. Similarly, peripheral B-cell counts showed significant correlations with selected coagulation parameters, most consistently with aPTT and PTT-LA mix.

Posttreatment VDRL titers showed strong positive correlations with posttreatment BAFF concentrations (ρ = 0.93, *p* < 0.001) and B-cell counts (ρ = 0.73, *p* < 0.001). In addition, posttreatment VDRL titers correlated with several phospholipid-dependent coagulation assays, including PTT-LA mix (ρ = 0.64, *p* < 0.001), aPTT (ρ = 0.54, *p* = 0.002), and LA1/LA2 ratio (ρ = 0.48, *p* = 0.012). Baseline VDRL titers also correlated with baseline BAFF levels (ρ = 0.76, *p* < 0.001).

Taken together, these findings indicate that persistent serological activity is associated with increased BAFF/B-cell–related immune activation and with the magnitude of phospholipid-dependent clotting-time prolongation.

### 2.5. High-Value Burden Relative to Healthy Controls

To further assess the consistency of phospholipid-dependent abnormalities, we compared the frequency of values exceeding the 99th percentile derived from healthy controls (Supplementary Table S1). Across all tested parameters (aPTT, LA1, LA2, LA1/LA2 ratio, PTT-LA, and PTT-LA mix), serofast patients exhibited a higher prevalence of results above the control-derived cutoff compared with non-serofast individuals (*p* < 0.05 for most tests).

These findings indicate that even in the absence of formal lupus anticoagulant positivity, a subset of serofast patients demonstrated values within a subclinical “high-value” range, suggesting subtle yet consistent phospholipid-dependent alterations following syphilis treatment.

### 2.6. Predictive Modeling of Serofast Status

To explore whether posttreatment phospholipid-dependent coagulation parameters were associated with persistent serological reactivity, logistic regression models were constructed using posttreatment aPTT, LA1, LA2, and PTT-LA mix as predictors (Model 1). An extended model additionally included age and sex (Model 2), and a further model incorporated baseline BAFF concentration (Model 3).

As shown in Supplementary Table S2 and Supplementary Figure S1A–C, all models demonstrated high apparent discriminatory performance. Model 1 yielded an apparent area under the receiver operating characteristic curve (AUC) of 0.99. Addition of age and sex (Model 2) resulted in an apparent AUC of 1.00, while incorporation of baseline BAFF (Model 3) preserved this high apparent performance and provided a biologically relevant immune-related predictor.

Among individual predictors, posttreatment aPTT, LA1, and PTT-LA mix showed the strongest associations with serofast status, whereas LA2 was not a consistent independent predictor across models. The inclusion of baseline BAFF further supported the link between B-cell activation and phospholipid-dependent coagulation abnormalities.

## 3. Discussion

The serofast state remains one of the most clinically challenging and poorly understood phenomena in the management of syphilis. Despite appropriate antibiotic therapy, a substantial proportion of patients fail to achieve the expected serological response, and the interpretation of persistent non-treponemal reactivity remains uncertain [2,3]. This ambiguity has direct clinical consequences, as it frequently leads to repeated antibiotic treatment despite limited evidence of benefit [2,4,5,6].

Importantly, before attributing persistent seroreactivity to post-infectious immune mechanisms, asymptomatic neurosyphilis (ANS) must be actively considered and excluded. In our cohort, 11 of 36 patients (30.6%) demonstrated an inadequate serological response at 6 months. Cerebrospinal fluid (CSF) examination identified ANS in 3 of these individuals (27.3%), who were subsequently excluded from further analysis. Although this proportion is moderate, it is clinically highly relevant. Previous studies have reported similar rates of ANS among serological non-responders, ranging from approximately 30% to 35% [19,20]. Moreover, untreated ANS may progress to symptomatic neurosyphilis in a substantial proportion of patients, underscoring the importance of early identification [19]. At the same time, routine retreatment of serofast patients without prior CSF evaluation has shown limited clinical benefit [20]. Currently, differentiation between ANS and a benign serofast state relies on invasive CSF examination, although emerging metabolomic approaches suggest that non-invasive biomarkers may become available in the future [21]. Taken together, these observations emphasize that failure to achieve an adequate decline in non-treponemal titers should prompt consideration of lumbar puncture to exclude ANS before further therapeutic decisions are made.

In the present study, we approached this problem from a different perspective by integrating coagulation, immunological, and serological parameters. We demonstrate that patients with serofast status exhibit a consistent pattern of phospholipid-dependent coagulation abnormalities, accompanied by increased BAFF levels and elevated B-cell counts. Notably, these alterations were coherent across multiple assays and strongly correlated with posttreatment VDRL titers, suggesting that persistent seroreactivity is linked to a coordinated immune–coagulation response rather than assay variability.

To our knowledge, this is among the first studies to systematically evaluate phospholipid-dependent coagulation profiles in serofast syphilis. The observed prolongation of aPTT-LA, LA1, LA2, LA1/LA2 ratio, and PTT-LA mix indicates functional interference with phospholipid-dependent clotting reactions. Although only a minority of patients fulfilled formal criteria for lupus anticoagulant positivity, the consistency of these findings across assays may suggest the presence of low-level phospholipid-reactive immune activity exerting subtle functional effects.

These observations extend our previous work on immune dysregulation in serofast syphilis. Earlier studies demonstrated that serofast patients exhibit features of altered immune regulation, including increased prevalence of antinuclear antibodies and shifts toward immunoregulatory cytokine profiles [7]. In addition, genetic and functional studies have implicated impaired Th1 responses, including reduced interferon-γ signaling, as well as enhanced anti-inflammatory pathways such as IL-10, in determining serological outcomes [7,8]. Together, these findings support a model in which the serofast state reflects a host-dependent immunological phenotype rather than persistent infection.

The present study adds a new dimension to this model by linking immune dysregulation with phospholipid-dependent coagulation abnormalities. Non-treponemal tests such as VDRL and RPR detect antibodies directed against cardiolipin, which are not specific to *Treponema pallidum* and are also characteristic of antiphospholipid autoimmunity [9,10,22,23]. Our findings suggest that, in serofast patients, persistent VDRL reactivity may partly reflect infection-induced antiphospholipid antibodies rather than ongoing microbial activity.

This interpretation is further supported by parallels with other infectious diseases. Transient antiphospholipid antibodies and lupus anticoagulant–related abnormalities have been described in conditions such as COVID-19, HIV infection, and hepatitis C, where they are thought to arise from infection-driven immune activation and molecular mimicry [13,14,15]. In this context, the serofast state may represent a similar phenomenon in which infection triggers a sustained but dysregulated humoral immune response.

**Figure 2:**
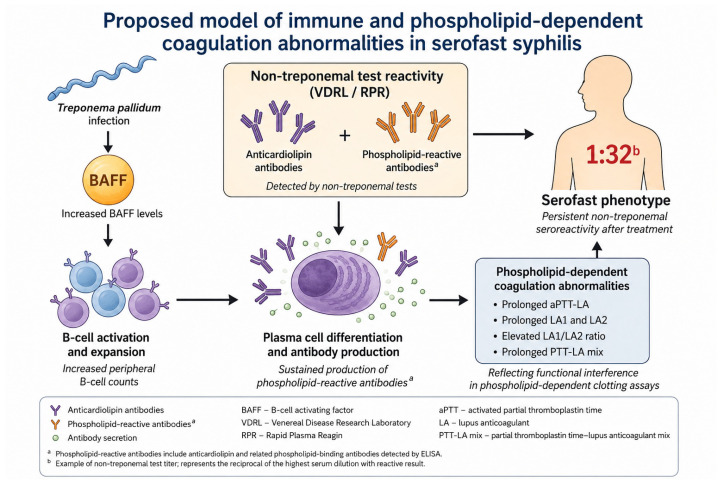
Proposed model of immune and phospholipid-dependent coagulation abnormalities in serofast syphilis. Schematic representation of the proposed immune–coagulation interactions associated with persistent non-treponemal seroreactivity after treatment of early syphilis. *Treponema pallidum* infection may promote B-cell activation and increased BAFF signaling, potentially facilitating the generation and persistence of phospholipid-reactive antibodies. These immune responses may contribute to prolonged phospholipid-dependent coagulation assays and persistent non-treponemal test reactivity observed in serofast patients. The model is hypothesis-generating and based on associations observed in the present study.

The observed elevation of BAFF levels and expansion of peripheral B-cell populations in serofast patients may represent a biologically plausible link between infection-associated immune activation and persistent phospholipid-reactive antibody responses. A schematic summary of the proposed immune–coagulation interactions underlying the serofast phenotype is presented in Figure 2. BAFF is a key regulator of B-cell survival and is known to promote the persistence of autoreactive B-cell clones in autoimmune diseases [17,18]. In the setting of infection, prolonged BAFF signaling may facilitate the generation and maintenance of cross-reactive antiphospholipid antibodies, which could contribute both to phospholipid-dependent coagulation abnormalities and to persistent non-treponemal seroreactivity.

From a clinical perspective, these findings offer a potential explanation for the limited efficacy of repeated antibiotic therapy in serofast patients. If persistent VDRL positivity in at least a subset of serofast patients reflects immune-mediated mechanisms rather than viable *T. pallidum*, further antimicrobial treatment may have limited impact on serological outcomes. Instead, the serofast state may represent a post-infectious immune phenomenon, analogous to other infection-triggered autoimmune responses.

Importantly, our study does not establish causality, and alternative explanations cannot be fully excluded. It is possible that pre-existing or subclinical immune differences predispose certain individuals to both altered coagulation profiles and persistent serological responses. Nevertheless, the consistency of findings across multiple independent assays and their strong correlation with VDRL titers argue against random variability.

Several limitations should be acknowledged. The sample size was modest, and the findings require validation in larger cohorts. Lupus anticoagulant testing was performed at two time points without repeat confirmation at 12 weeks, as recommended by ISTH guidelines. Additionally, we cannot fully exclude the influence of confounding factors, including inflammatory or metabolic conditions, on phospholipid-dependent assays. The absence of comparator groups with autoimmune or other infectious diseases also limits our ability to determine whether the observed phospholipid-dependent abnormalities are specific to syphilis or represent a broader infection-associated immune phenomenon. Despite these limitations, the coherence of the observed immune and coagulation patterns strengthens the biological plausibility of our findings.

In summary, we demonstrate that the serofast state after early syphilis treatment is associated with a consistent pattern of B-cell activation and phospholipid-dependent coagulation abnormalities. These findings support the concept that persistent non-treponemal seroreactivity, after exclusion of neurosyphilis, may reflect an infection-triggered immune phenotype rather than ongoing infection.

## 4. Materials and Methods

### 4.1. Study Design

This was a prospective observational study conducted between 2024 and 2025 at the Clinical Department of Dermatology in Zabrze, Medical University of Silesia in Katowice, Poland. All consecutive patients meeting predefined eligibility criteria and diagnosed with early syphilis were invited to participate, without additional selection.

### 4.2. Patient Characteristics and Eligibility Criteria

Patients were eligible if they were aged ≥18 years and presented with a first episode of early syphilis (secondary or early latent stage). Disease staging followed the Centers for Disease Control and Prevention (CDC) criteria [24] and was based on clinical history, physical examination, and laboratory findings.

Early latent syphilis was diagnosed when both treponemal and non-treponemal tests were positive and at least one of the following criteria was met within the preceding 12 months: (i) documented serologic relapse, (ii) a ≥4-fold increase in non-treponemal titer, or (iii) presumptive clinical evidence of primary or secondary syphilis.

Exclusion criteria included antimicrobial or immunosuppressive therapy within six months prior to enrollment, HIV infection, a history of chronic inflammatory or autoimmune disorders, and the use of anticoagulant or antiplatelet agents within at least six weeks preceding study entry.

All enrolled patients received a single intramuscular dose of benzathine penicillin G (2.4 million units) in accordance with current treatment guidelines. Baseline demographic and clinical data (age, sex, and syphilis stage) were recorded.

At six months post-treatment, patients were initially evaluated for serological response based on VDRL titers. Individuals who failed to achieve a ≥4-fold decline in titer were considered to have an inadequate serological response and were further evaluated for neurosyphilis.

In all such cases, cerebrospinal fluid (CSF) examination was performed. The diagnosis of neurosyphilis was established according to CDC criteria, including reactive CSF VDRL and/or pleocytosis (≥5 cells/µL) with elevated protein concentration.

A total of 11 patients demonstrated an inadequate serological response at 6 months. Among them, 3 were diagnosed with neurosyphilis and were excluded from further analysis. The remaining 8 patients, in whom central nervous system infection was excluded, were classified as the serofast group.

Patients who achieved a ≥4-fold decline in VDRL titer were classified as the serologically cured group, in accordance with previously published criteria [3].

Consequently, the serofast group included in the present study consisted exclusively of individuals with persistent serological reactivity despite exclusion of central nervous system infection.

### 4.3. Sample Collection

Blood samples for serological and immunological analyses were collected at two time points:

(1) baseline (before antibiotic administration) and

(2) six months after treatment completion.

A control group of 11 healthy volunteers without a history of syphilis, autoimmune disease, or anticoagulant therapy was included for reference purposes. In these individuals, venous blood was collected once and analyzed using the same laboratory procedures.

Venous blood was drawn into 3.2% sodium citrate tubes (blood-to-citrate ratio 9:1). Platelet-poor plasma was prepared by double centrifugation (<10 × 10^9^/L platelets) and either analyzed within 4 h or stored at −80 °C until testing.

### 4.4. Coagulation Assays

Phospholipid-dependent coagulation tests were performed using reagents from Siemens Healthineers (Marburg, Germany) on an automated coagulation analyzer.

The following assays were included:

aPTT-LA: lupus anticoagulant–sensitive activated partial thromboplastin time using low phospholipid concentration; dRVVT screen and confirm (LA1 and LA2): LA1 used low phospholipid content, while LA2 contained high phospholipid concentration; the LA1/LA2 ratio was calculated; PTT-LA mix: performed on a 1:1 mixture of patient plasma and normal pooled plasma. Results were expressed in seconds (aPTT, LA1, LA2, PTT-LA mix) and as a ratio (LA1/LA2). Internal quality control included normal and lupus anticoagulant–positive reference plasmas. Although formal lupus anticoagulant positivity according to ISTH criteria was observed in only two patients, all parameters were analyzed as continuous variables to capture subclinical phospholipid-dependent coagulation abnormalities.

### 4.5. Antiphospholipid Antibody Assays

IgG and IgM anticardiolipin antibodies (aCL) and anti-β_2_ glycoprotein I antibodies (anti-β_2_GPI) were quantified using standardized enzyme-linked immunosorbent assays (ELISA) (e.g., QUANTA Lite^®^ ELISA kits (Inova Diagnostics, a DiaSorin company, San Diego, CA, USA).

Results were expressed in GPL (IgG), MPL (IgM), or standardized SGU/SMU units. Antibody levels ≥40 GPL/MPL or above the 99th percentile of healthy controls were considered positive, in accordance with international consensus criteria.

Both assays were performed at baseline and six months post-treatment.

### 4.6. Immunological Assays

Peripheral blood B lymphocyte counts were determined by flow cytometry using standard immunophenotyping protocols with CD19 as a B-cell marker.

Absolute B-cell counts were calculated using concurrent hematological parameters and expressed as ×10^3^/µL.

Serum BAFF concentrations were measured using commercially available ELISA kits (Thermo Fisher Scientific, Waltham, MA, USA), with an analytical range of 0.31–20 ng/mL. All samples were analyzed in duplicate.

### 4.7. Syphilis Serology

Serological testing included both non-treponemal and treponemal assays:

Venereal Disease Research Laboratory (VDRL) test: performed using a standardized antigen suspension (e.g., BD Macro-Vue™ BD Diagnostics, Franklin Lakes, NJ, USA); titers were determined by serial twofold dilutions. *Treponema pallidum* hemagglutination assay (TPHA): performed using a commercial kit (e.g., TPHA test, Bio-Rad, Marnes-la-Coquette, France). Positive results in both assays were required for inclusion.

### 4.8. Statistical Analysis

Continuous variables were expressed as median (min–max), and comparisons between groups were performed using the Mann–Whitney U test. Categorical variables were compared using the χ^2^ or Fisher’s exact test.

Multiple testing correction was performed using the Benjamini–Hochberg false discovery rate.

To evaluate longitudinal changes and differences between groups over time, a two-way model including group, time, and group × time interaction was applied for selected immune parameters (BAFF levels and B-cell counts), corresponding to a difference-in-differences framework.

Associations between variables were assessed using Spearman rank correlation coefficients (ρ), and results were visualized using a correlation heatmap. Effect sizes were calculated using Cliff’s delta.

Exploratory logistic regression models were constructed to assess whether coagulation and immune parameters were associated with serofast status. Model performance was evaluated using receiver operating characteristic (ROC) curves and the area under the curve (AUC).

Given the limited sample size, these analyses were considered exploratory and were not internally validated; therefore, model performance estimates should be interpreted with caution.

All analyses were performed using R (version 4.3.2) and GraphPad Prism (version 10.0).

## Figures and Tables

**Table 1:** Baseline and posttreatment clinical and laboratory characteristics of patients with early syphilis, stratified by serological response.

	Serofast Group, n = 8	Non-Serofast Group, n = 25	*p* Value
age	31.5 (28–45)	34 (22–69)	0.22
females; n (%)	2 (25)	7 (28)	0.87
secondary syphilis; n (%)	4 (50)	11 (44)	0.77
early latent syphilis; n (%)	4 (50)	14 (56)	0.77
			
baseline VDRL	64 (8–128)	64 (2–256)	0.44
baseline leukocytes count; ×10^3^/uL	6.7 (4.2–10.7)	7 (3.8–9.0)	0.77
baseline lymphocytes count; ×10^3^/uL	1.3 (0.6–1.8)	1.6 (0.9–2.8)	0.04
baseline B cells count; ×10^3^/uL	293.5 (205–606)	215.5 (72–582)	0.03
baseline BAFF levels; ng/mL	2.6 (1.5–3.9)	1.6 (0.3–3.1)	0.03
baseline platelets count; ×10^3^/uL	260 (207–340)	259 (149–460)	0.77
baseline CRP levels; mg/dL	2.5 (1.3–33.0)	6.2 (0–23.4)	0.58
baseline ANA titer	0 (0–160)	0 (0–160)	0.64
			
posttreatment VDRL	48 (8–128)	4 (0–16)	0.00001
posttreatment leukocytes count; ×10^3^/uL	7.2 (4.1–11.0)	6.2 (3.5–8.5)	0.13
posttreatment lymphocytes count; ×10^3^/uL	2.0 (1.6–2.2)	1.9 (1.2–3.1)	0.82
posttreatment B cells count; ×10^3^/uL	380 (297–497)	216 (109–584)	0.0.0008
posttreatment BAFF levels; ng/mL	1.7 (0.9–2.9)	0.44 (0.3–2.3)	0.002
posttreatment platelets count; ×10^3^/uL	267 (210–323)	248 (137–350)	0.56
posttreatment CRP levels; mg/dL	1.9 (0–3.5)	1.0 (0–22.6)	1.0
posttreatment ANA titer	0 (0–320)	0 (0–160)	0.59

VDRL—Venereal Disease Research Laboratory; BAFF—B-cell activating factor belonging to the TNF family; CRP—C-reactive protein; ANA—antinuclear antibody.

**Table 2:** Phospholipid-dependent coagulation parameters and antiphospholipid antibody profiles in patients with early syphilis.

	Serofast Group, n = 8	Non-Serofast Group, n = 25	*p* Value
baseline aCL IgG; U/mL	17.5 (0–61.7)	11.5 (0–120)	0.9
baseline aCL IgM; U/mL	22.9 (0–67.5)	10.2 (0–120)	0.9
baseline anti-β_2_GPI; U/mL	2.78 (2–4.2)	2.23 (2–3.8)	0.8
baseline aPTT; s	36.2 (33.2–46.2)	30.3 (26.0–41.3)	0.004
baseline LA1; s	44.6 (38.7–56.2)	40.2 (29.5–59.5)	0.08
baseline LA2; s	35.0 (34.1–40.4)	35.0 (32.2–40.2)	0.17
baseline LA1/LA2	1.04 (1.0–1.4)	1.0 (1.0–1.5)	0.37
baseline PTT-LA mix; s	36.3 (35.0–52.2)	35.0 (33.5–51.3)	0.48
			
posttreatment aCL IgG; U/mL	0.0 (0.0–6.2)	0.0 (0.0–5.4)	0.48
posttreatment aCL IgM; U/mL	0.0 (0.0–6.7)	0.0 (0.0–14.0)	0.66
posttreatment anti-β_2_GPI; U/mL	2.87 (2–3.7)	2.45 (2–4.1)	0.87
posttreatment aPTT; s	37.8 (31.1–49.0)	26.0 (25.0–32.5)	0.00001
posttreatment LA1; s	48.5 (40.6–62.6)	36.7 (28.0–48.2)	0.002
posttreatment LA2; s	35.9 (35.0–39.0)	35.0 (35.0–36.3)	0.0002
posttreatment LA1/LA2	1.4 (1.0–1.7)	1.0 (1.0–1.3)	0.0001
posttreatment PTT-LA mix; s	42.9 (35.0–57.3)	35.0 (35.0–42.1)	0.00001

aCL IgG—anti-cardiolipin immunoglobulin G antibodies; aCL IgM—anti-cardiolipin immunoglobulin M antibodies; anti-β_2_GPI—anti–beta-2 glycoprotein I antibodies; aPTT—activated partial thromboplastin time; LA1—dilute Russell viper venom test (screen, low phospholipid concentration); LA2—dilute Russell viper venom test (confirm, high phospholipid concentration); PTT-LA mix—phospholipid-dependent partial thromboplastin time mixing study. Effect sizes (Cliff’s delta) and FDR-adjusted *p*-values (q_BH_) are reported in the text.

## Data Availability

The original contributions presented in this study are included in the article/Supplementary Materials. Further inquiries can be directed to the corresponding author.

## Supplementary Materials

The following supporting information can be downloaded at: https://www.mdpi.com/article/10.3390/ijms27114954/s1.
