# Supplementary material for: Serofast Syphilis Is Associated with Phospholipid-Dependent Coagulation Abnormalities and B-Cell Activation Following Treatment
Source: Int J Mol Sci. 2026 May 29;27(11):4954. doi: 10.3390/ijms27114954 (PMC13256180; doi:10.3390/ijms27114954)
Supplement: Supplementary file 1 [file ijms-27-04954-s001.zip › ijms-4189367-supplementary.pdf]

Supplementary Figure S1. Receiver operating characteristic (ROC) curves for exploratory logistic regression models predicting serofast status based on phospholipid-dependent coagulation parameters and immune markers.

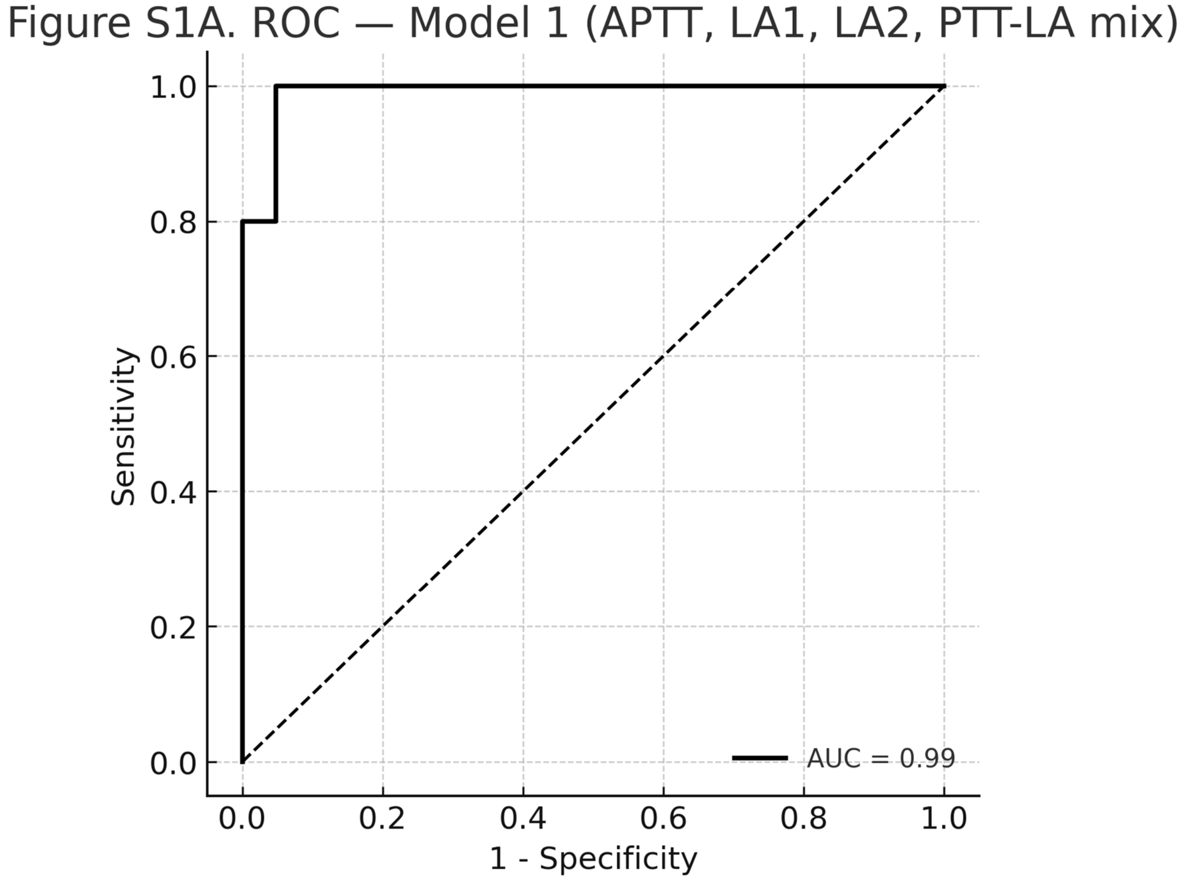

Figure S1A. Model 1.

Receiver operating characteristic (ROC) curve for the logistic regression model including posttreatment phospholipid-dependent coagulation parameters (aPTT-LA, LA1, LA2, and PTT-LA mix). The apparent area under the curve (AUC) was 0.99.

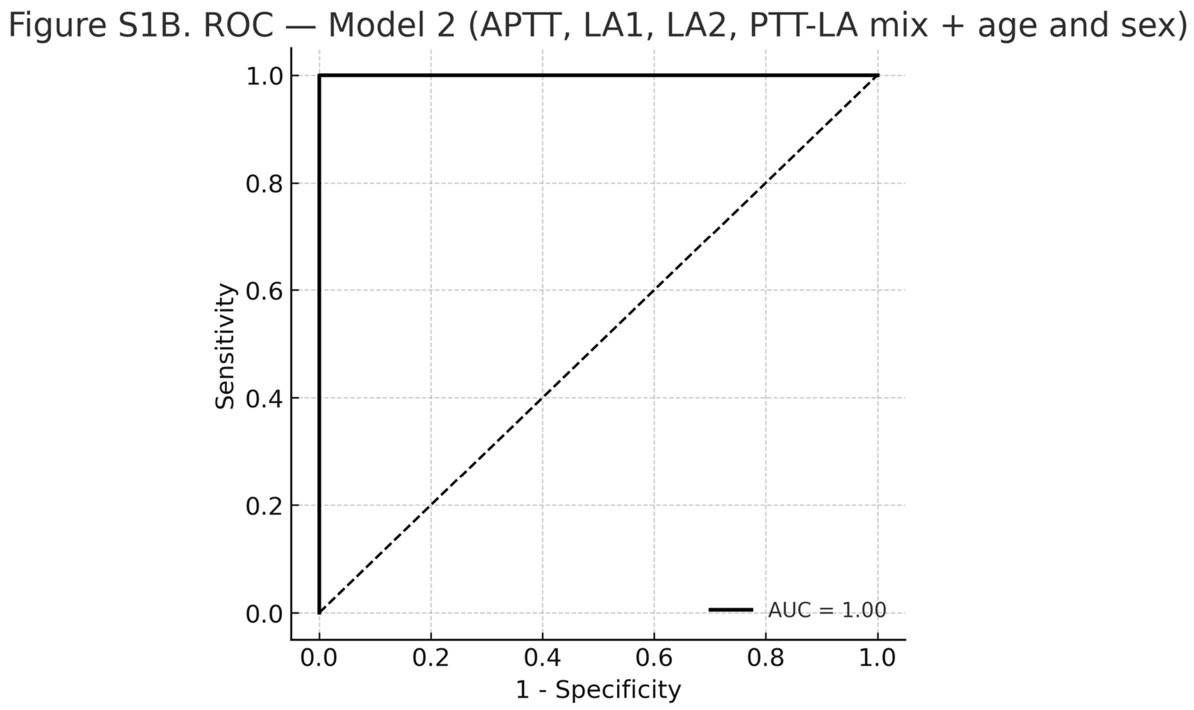

Figure S1B. Model 2.

Receiver operating characteristic (ROC) curve for the logistic regression model including posttreatment phospholipid-dependent coagulation parameters (aPTT-LA, LA1, LA2, and PTT-LA mix), adjusted for age and sex. The apparent AUC was 1.00.

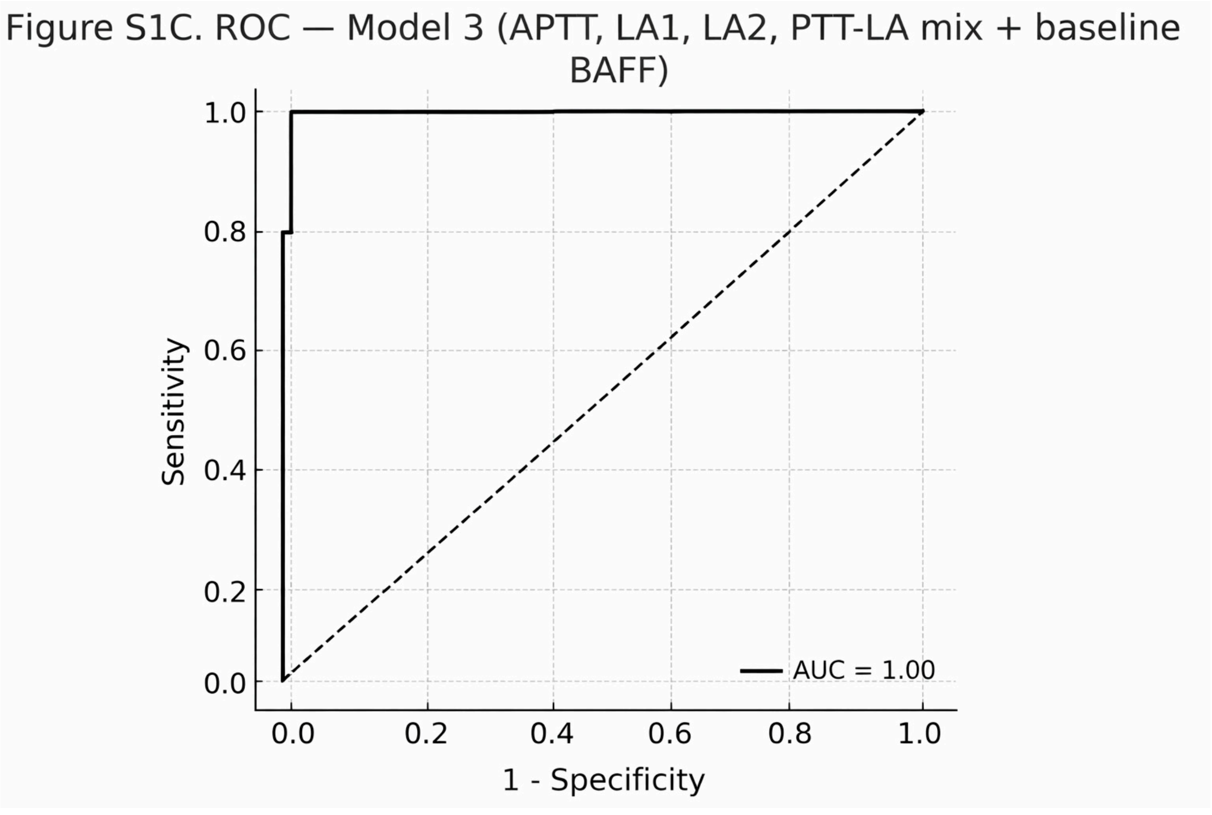

Figure S1C. Model 3.

Receiver operating characteristic (ROC) curve for the extended logistic regression model including posttreatment phospholipid-dependent coagulation parameters (aPTT-LA, LA1, LA2, and PTT-LA mix) together with baseline BAFF concentration. The apparent AUC was 1.00.

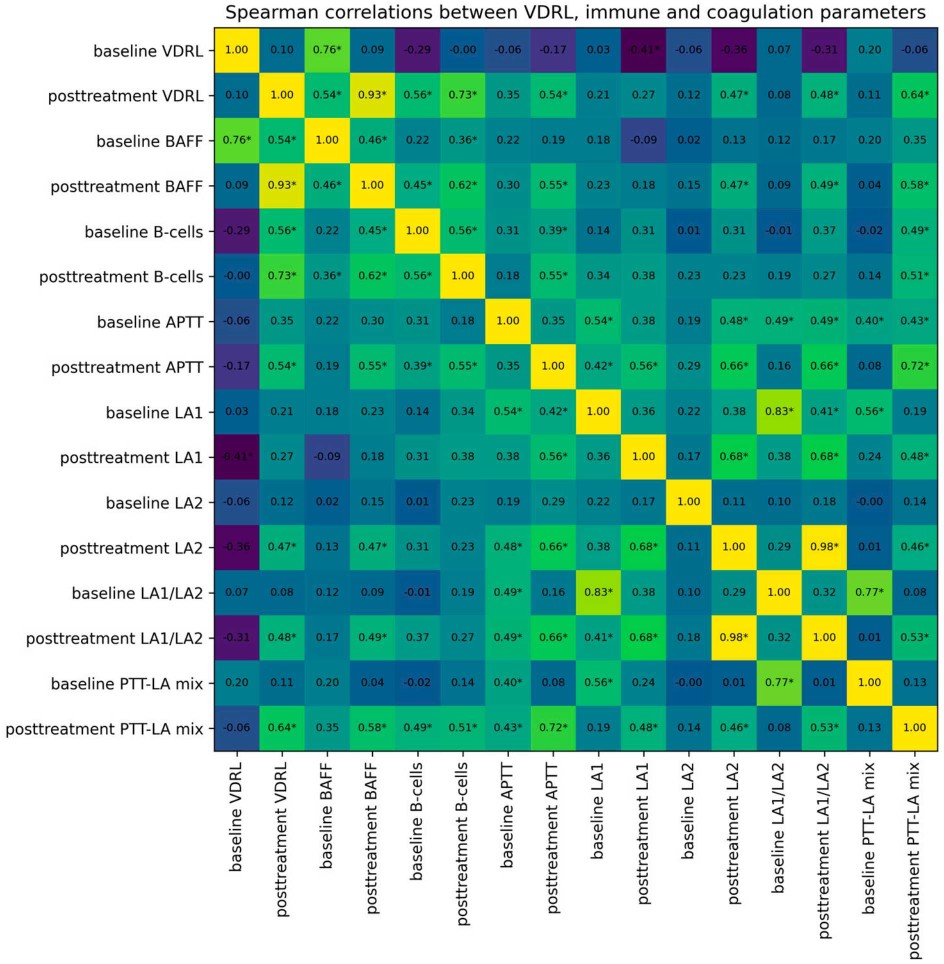

Supplementary Figure S2. Correlation heatmap of serological, immune, and phospholipid-dependent coagulation parameters.

Heatmap showing Spearman correlation coefficients (ϱ) between VDRL titers, BAFF concentrations, peripheral B-cell counts, and phospholipid-dependent coagulation parameters measured at baseline and 6 months after treatment.

Variables included baseline and posttreatment values of VDRL, BAFF, B-cell counts, activated partial thromboplastin time (aPTT-LA), LA1, LA2, LA1/LA2 ratio, and PTT-LA mix. Each cell represents the pairwise Spearman correlation coefficient between variables.

Asterisks (*) indicate statistically significant correlations ($p < 0.05$). Warmer colors denote positive correlations, whereas cooler colors denote negative correlations.

Supplementary Table S1. Proportion of patients exceeding the 99th percentile of phospholipid-dependent coagulation parameters relative to healthy controls

Parameter	99th percentile (controls)	% > cutoff (non-serofast)	% > cutoff (serofast)	Fisher's p (serofast > non-serofast)
aPTT	39.8	4.3	27.3	0.046
LA1	46.2	8.7	36.4	0.041
LA2	54.7	0	27.3	0.020
LA1/LA2 ratio	1.30	4.3	27.3	0.046
PTT-LA mix	39.6	4.3	27.3	0.046

aPTT — activated partial thromboplastin time; LA1 — dilute Russell viper venom test (screen, low phospholipid concentration); LA2 — dilute Russell viper venom test (confirm, high phospholipid concentration); PTT-LA mix — phospholipid-dependent partial thromboplastin time mixing study.

Proportion of patients exceeding the 99th percentile derived from healthy controls for phospholipid-dependent coagulation parameters. The 99th percentile thresholds were established in the control cohort. The table shows the percentage of patients with posttreatment values above this cutoff in each group (serofast vs non-serofast). Fisher's exact test (one-sided, alternative = greater) was used to assess whether the proportion of "high-value" results was significantly higher among serofast individuals.

Supplementary Table S2. Logistic regression models predicting serofast status

Model 1 (aPTT, LA1, LA2, PTT-LA mix)				
Predictor (per 1 SD)	OR	95% CI (OR)	p-value	AUC (95% CI)
aPTT	2.70	1.76–3.68	<0.001	0.99 (0.95-1.00)
LA1	2.32	1.18–4.41	0.003	
LA2	1.49	0.57–2.81	0.386	
PTT-LA mix	2.15	1.00–3.88	0.019	
Model 2 (aPTT, LA1, LA2, PTT-LA mix + age and sex)				
Predictor (per 1 SD)	OR	95% CI (OR)	p-value	AUC (95% CI)
aPTT	2.48	1.73–3.04	<0.001	1.00 (0.97-1.00)
LA1	2.21	1.23–3.40	<0.001	
LA2	1.88	0.80–2.83	0.336	
PTT-LA mix	1.89	1.00–3.32	0.032	
Age	0.84	0.61–1.18	0.360	
Females	2.11	0.88–3.10	0.255	
Model 3 (aPTT, LA1, LA2, PTT-LA mix + baseline BAFF)				
Predictor (per 1 SD)	OR	95% CI (OR)	p-value	AUC (95% CI)
aPTT	2.41	1.62-3.10	<0.001	1.00 (0.97-1.00)
LA1	2.16	1.14-3.21	0.002	
LA2	1.67	0.7-2.74	0.342	
PTT-LA mix	1.92	1.02-3.01	0.028	
baseline BAFF	1.84	1.11-2.74	0.017	

OR, odds ratio; CI, confidence interval; AUC, area under the receiver operating characteristic curve; SD, standard deviation; aPTT — activated partial thromboplastin time; LA1 — dilute Russell viper venom test (screen, low phospholipid concentration); LA2 — dilute Russell viper venom test (confirm, high phospholipid concentration); PTT-LA mix — phospholipid-dependent partial thromboplastin time mixing study, BAFF – B-cell activating factor.
